# Extracellular Vesicles From Mesenchymal Umbilical Cord Cells Exert Protection Against Oxidative Stress and Fibrosis in a Rat Model of Bronchopulmonary Dysplasia

**DOI:** 10.1093/stcltm/szad070

**Published:** 2023-11-15

**Authors:** Paola Bisaccia, Fabio Magarotto, Stefania D’Agostino, Arben Dedja, Silvia Barbon, Diego Guidolin, Cristina Liboni, Roberta Angioni, Giada De Lazzari, Federico Caicci, Antonella Viola, Marcin Jurga, Gabrielis Kundrotas, Dimitri Stevens, Domenico Mancuso, Elisabetta Gramegna, Bruno Seitaj, Rudra Kashyap, Beatrice De Vos, Veronica Macchi, Eugenio Baraldi, Andrea Porzionato, Raffaele De Caro, Maurizio Muraca, Michela Pozzobon

**Affiliations:** Department of Women’s and Children’s Health, University of Padova, Padova, Italy; Institute of Pediatric Research Città della Speranza, Padova, Italy; Department of Women’s and Children’s Health, University of Padova, Padova, Italy; Institute of Pediatric Research Città della Speranza, Padova, Italy; Department of Women’s and Children’s Health, University of Padova, Padova, Italy; Institute of Pediatric Research Città della Speranza, Padova, Italy; Department of Cardiac, Thoracic and Vascular Sciences and Public Health, University of Padova, Padova, Italy; Department of Neuroscience, University of Padova, Padova, Italy; Department of Neuroscience, University of Padova, Padova, Italy; Institute of Pediatric Research Città della Speranza, Padova, Italy; Department of Biomedical Sciences, University of Padova, Padova, Italy; Institute of Pediatric Research Città della Speranza, Padova, Italy; Department of Biomedical Sciences, University of Padova, Padova, Italy; Department of Women’s and Children’s Health, University of Padova, Padova, Italy; Institute of Pediatric Research Città della Speranza, Padova, Italy; Department of Biology, University of Padua, Padova, Italy; Institute of Pediatric Research Città della Speranza, Padova, Italy; Department of Biomedical Sciences, University of Padova, Padova, Italy; EXO Biologics SA, Liège, Belgium; EXO Biologics SA, Liège, Belgium; EXO Biologics SA, Liège, Belgium; EXO Biologics SA, Liège, Belgium; EXO Biologics SA, Liège, Belgium; EXO Biologics SA, Liège, Belgium; EXO Biologics SA, Liège, Belgium; EXO Biologics SA, Liège, Belgium; Department of Neuroscience, University of Padova, Padova, Italy; Department of Women’s and Children’s Health, University of Padova, Padova, Italy; Institute of Pediatric Research Città della Speranza, Padova, Italy; Department of Neuroscience, University of Padova, Padova, Italy; Department of Neuroscience, University of Padova, Padova, Italy; Department of Women’s and Children’s Health, University of Padova, Padova, Italy; Institute of Pediatric Research Città della Speranza, Padova, Italy; Department of Women’s and Children’s Health, University of Padova, Padova, Italy; Institute of Pediatric Research Città della Speranza, Padova, Italy

**Keywords:** extracellular vesicles, bronchopulmonary dysplasia, macrophages, lung fibrosis, oxidative stress

## Abstract

Oxidative stress and fibrosis are important stress responses that characterize bronchopulmonary dysplasia (BPD), a disease for which only a therapy but not a cure has been developed. In this work, we investigated the effects of mesenchymal stromal cells-derived extracellular vesicles (MSC-EVs) on lung and brain compartment in an animal model of hyperoxia-induced BPD. Rat pups were intratracheally injected with MSC-EVs produced by human umbilical cord-derived MSC, following the Good Manufacturing Practice-grade (GMP-grade). After evaluating biodistribution of labelled MSC-EVs in rat pups left in normoxia and hyperoxia, oxidative stress and fibrosis investigation were performed. Oxidative stress protection by MSC-EVs treatment was proved both in lung and in brain. The lung epithelial compartment ameliorated glycosaminoglycan and surfactant protein expression in MSC-EVs-injected rat pups compared to untreated animals. Pups under hyperoxia exhibited a fibrotic phenotype in lungs shown by increased collagen deposition and also expression of profibrotic genes. Both parameters were reduced by treatment with MSC-EVs. We established an in vitro model of fibrosis and another of oxidative stress, and we proved that MSC-EVs suppressed the induction of αSMA, influencing collagen deposition and protecting from the oxidative stress. In conclusion, intratracheal administration of clinical-grade MSC-EVs protect from oxidative stress, improves pulmonary epithelial function, and counteracts the development of fibrosis. In the future, MSC-EVs could represent a new cure to prevent the development of BPD.

## Graphical Abstract

**Graphical Abstract:**
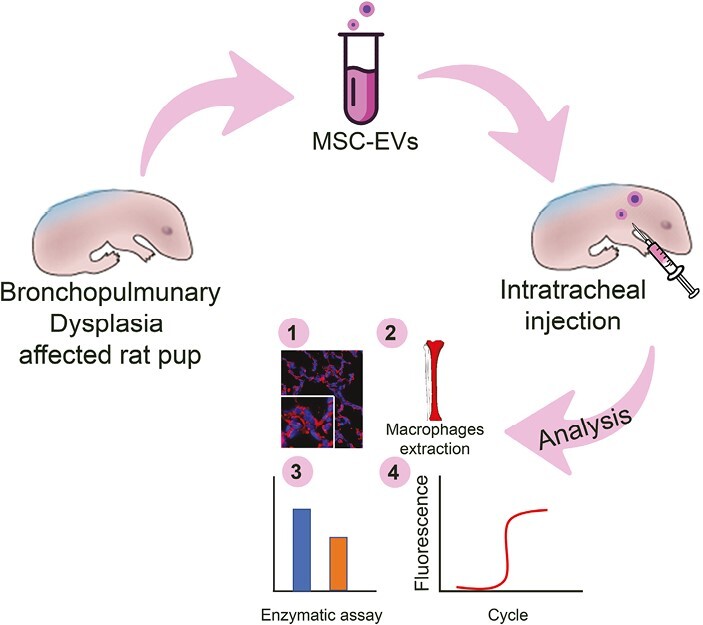
Clinical grade MSC-EVs were intratracheally injected in a rat model of broncopulmonary dysplasia. Fibrosis and oxidative stress have been analyzed in lung and brain without and with MSC-EVs.

**Significance Statement**

The present work comprehensively analyzed the effect of the MSC-EVs that our group have already proved to be effective in the rat model of BPD. We studied in vivo and in vitro the fibrosis process and the antioxidant action of the nanoparticles. To our knowledge, this is the first study that investigate in vivo and in vitro the path of fibrosis process and the antioxidant action of the nanoparticles.

## Introduction

Extracellular vesicles (EVs) are nanoparticles released by all types of cells, in particular those from mesenchymal stromal cells (MSC-EVs) have recently shown their role in regulating the balance between proinflammatory and anti-inflammatory cytokines,^1^ in modulating fibrosis and the functional tissue regeneration after damage.^2^

Recently, EVs have been studied as an effective tool against bronchopulmonary dysplasia (BPD).^3-6^This pathology affects premature babies and several factors, although not clear how they interact, have been implicated in the pathogenesis of BPD, such as oxygen, mechanical ventilation-mediated lung injury, infection/inflammation, and genetic risk factors.^7,8^ All premature babies’ lungs are exposed to supraphysiologic oxygen levels, and many infants are treated with supplemental inspired oxygen. Oxygen therapy in itself is a risk factor for BPD development and correlates with long-term respiratory symptoms. Thereby, the sublethal oxygen concentrations induce lung interstitial thickening and myofibroblast accumulation. At the same time, premature babies possess an undeveloped antioxidant system.

The production of Reactive Oxygen Species (ROS) is a physiological process that mediates cell-response to different stimuli, from cell homeostasis to organ development and pathophysiological signal transduction.^9,10^ An imbalance between ROS production and the ability of the antioxidant system to quickly detoxify the free radicals, results in oxidative stress^11^ that elicits cell death causing damage to DNA, proteins and lipids.

It is known that the impairment between ROS generated by the mechanical ventilation and the immature antioxidant system triggers and enhances the development of the BPD pathology.^11,12^ From the histological aspect, BPD is characterized by endothelial and epithelial cell damage, bronchial smooth muscle hypertrophy, interstitial fibrosis, and reduction of total number and surface area of alveoli.^13^ In lung epithelium, the alveolar type II (ATII) cells are the lung progenitor cells responsible of the repair producing surfactant and regeneration of alveolar type I (ATI) cells, which allow the gas exchange and are also quite susceptible to injury.^14^ Fibrosis and oxidative stress damage ATI. The 2 processes are strictly related with ROS activating TGFβ, a potent fibrotic factor, responsible to stimulate macrophages to become myofibroblasts that overproduce collagen.^15^ Importantly, macrophages are the main inflammatory cell type in BPD that release ROS and ROS released by them nourish oxidative processes.^16^ Beside lungs, also brain is affected in BPD. Cognitive deficits or behavioral problems during the later stages of development are documented.^17^

After these premises, the purpose of this work was to validate the effects of GMP-grade MSC-EVs against oxidative stress and fibrosis both in the lung and in the brain, in a rat model of BPD. In addition, to thoroughly analyze the fibrosis process, we investigated if the MSC-EVs blocked the change of phenotype of the macrophages toward myofibroblast in vitro.

## Materials and Methods

### Extracellular Vesicles

EVs were produced using human MSCs derived from umbilical cord tissue (UCT). The biological material was supplied by a certified Biobank (Biothèque Hospitalo-Universitaire de Liège) registered at the Belgian Federal Agency for Medicines and Health Products in accordance with the Belgian Law of December 19, 2008 concerning the Procurement and Use of Human Body Material with the aim at Human Medical Application or Scientific Research and its royal decrees, all as amended. The cord tissues were obtained and handled according to EU Directives 2004/23/EC and 2006/86/EC.

### Reagents and Consumables

All critical reagents which have direct contact with the biological material were GMP quality and did not contain any undefined product, xeno originated components and were not contaminated with the nanoparticles. Most of the reagents used for EVs manufacturing are proprietary information of the company.

### EV Production

#### Production of Mesenchymal Stromal Cells Intermediate

Approximately 40 cm of UCT was transported to the production lab and processed depending on the arrival time on the same day or on the next day (maximum 24 hours from the C-section). The cord slices were transferred to cryovials and cryopreserved with Via Freeze (Cytiva, VF_30001) electric controlled rate freezer. UCT cryovials were thawed, and the MSCs were enzymatically extracted from the slices of umbilical cord using different enzymes (company proprietary information). Digested slices were immediately placed into 2D culture plates to initiate expansion of MSCs. Defined medium (company proprietary information) guarantees very high homogeneity of the MSCs and EVs with no risk of contamination with unknown EVs, and proteins present in sera, platelet lysates, or other undefined components of culture media. During primary growth, the MSCs attached and started to proliferate forming colonies. After the first passage, cells continued proliferating as a typical monolayer culture of MSCs adherent to plastic (synthetic coating, company proprietary information). Cell viability below 80% at any passage terminated the production process. Cell detachment was achieved by incubation with combination of enzymes (company proprietary information). After centrifugation, the cell pellet was resuspended in DPBS with Ca/Mg and was ready for cell count and viability test. After cell harvesting, the MSCs are cryopreserved (company proprietary information). Cells phenotypes are tested twice, after 2D expansion and after 3D expansion, using a standard CD markers panel described for identification of MSCs in ATMPs manufacturing (BD Stemflow, Human MSC Analysis Kit).

#### Production of Extracellular Vesicles

MSC-EVs production was performed in 3D stirring bioreactor culture systems using microcarrier beads. MSCs, mixed with the microcarriers and defined culture medium, were inoculated into the bioreactor system. Bioreactor, beads, and culture media are proprietary information. This approach allowed very effective EVs production with multiple harvests and efficient large-scale manufacturing process. Supernatants containing MSC-EVs were collected, and fresh medium was replaced in the bioreactor every 24 hours using a peristaltic pump and semiautomated module of the MSC-EVs production. Bags with harvested supernatants were stored at 4°C-8°C until pooling in the next production step.

Pooled supernatants (from all harvests qualified for MSC-EVs production) were subsequently processed using semi-automated module of the MSC-EVs purification. Particle concentration and size were measured using nanoparticle tracking analysis (NTA) as critical in-process quality control test (IPC) in a pooled bag. In addition, a sample of pooled medium was taken for mycoplasma testing, and a sample of cell supernatant was collected for viral testing.

#### EV Concentration and Characterization

Tangential flow filter (TFF) was performed using cassettes with a 100 kDa molecular weight cutoff membrane. TFF cassette purity (nanoparticles free) was tested using NTA. 100 kDa TFF membrane filtration allows the impurities to pass the membrane into the permeate (waste). Of note, the defined culture medium components were smaller than 100 kDa and could not be accumulated in the retentate during FTT filtration. EVs were larger than 100 kDa and could be concentrated in the retentate. An additional single wash step was performed after preconcentration using saline. After washing, the EVs were reconcentrated by TFF filtration, and NTA was performed to confirm a target concentration of 1.2E+11 particles/mL used in this project. Subsequently, EVs were removed from the FTT cassette and aliquoted. The vials with EVs were transferred into cryoboxes and frozen. EVs were ready to use immediately after thawing.

The EVs batch used in this project was tested using nanoparticle tracking (NTA) ZetaView, PMX220 TWIN (Particle Metrics).

Mean particle size was 106 nm (normal distribution), and the concentration was 1.2E + 11 particles/mL.

#### Phenotypic Characterization

MACSPlex exosome kit (Miltenyi Biotech) was used for antigen characterization (EV-tetraspanin markers CD9, CD63, CD81 were detected).

#### CryoTEM

The experiments were performed using a freshly thawed EVs (undiluted and diluted 1 to 10 with injection-grade saline). The sample was prepared following the protocol described in Delgado et al (2019).^18^ Analysis was performed with the instrument Tecnai Arctica-Transmission Electron Microscope by FEI Company. Total 311 EVs were counted and analyzed in 271 images captured. The images provided (as TIF and MRC files) were analyzed using the Fiji extension for ImageJ (freeware).

### Biodistribution

The study was approved by the Local Ethical Committee and received Ministerial Authorization (n°616/2016-PR released on June 17, 2016). MSC-EVs were stained with the lipophilic fluorescent marker DiR (1,1ʹ-dioctadecyl-3,3,3ʹ,3ʹ-tetramethylindotricarbocyanine iodide, Thermo Fisher Scientific), to assess MSC-EVs biodistribution after in vivo intratracheal (IT) injection following the already described procedure.^2^ Briefly, after EV-DiR and PBS-DiR (control) staining, centrifugation at 4°C at 2000*g* with the 100 kDa filters (Amicon-Merck Millipore) and retention of EV-DiR and PBS-DiR (150-200 μL), NTA analysis confirmed the absence of detectable particles (below 1E + 8 particles/mL) in PBS-DiR (Supplementary 2). Twenty pups, 7 days old, grown in normoxia (21% oxygen) and 20 pups, 7 days old, grown in hyperoxia (60% oxygen) were anesthetized with 3% isofluorane for 5 minutes and were divided in 4 groups, 2 in normoxia and 2 in hyperoxia distributed as follows: PBS-DiR (IT injection 50μL/ rat) was injected in 10 pups grown in normoxia and in 10 pups left in hyperoxia, EV-DiR (1E + 9 MSC-EVs, diluted in PBS 50μL/rat) was injected in 10 pups grown in normoxia and in 10 pups left in hyperoxia. Sacrifice was performed 3 hours and 24 hours post-IT treatment. For each time point, in normoxia and hyperoxia, 5 rats with MSC-EVs-DiR and 5 rats with PBS-DiR were sacrificed. After sacrifice, from each pup, organs (lungs, brain, inguinal and axillary lymph nodes, spleen, liver, kidney, heart, intestine were removed and snap frozen in liquid nitrogen, until analysis. After weighting each organ, 500 μL of homogenization buffer was added in safe seal tubes (Sarstedt), and tissue homogenate was obtained (tissue lyser2- Qiagen). Homogenization buffer was used as already described.^2^ The Ensight fluorimeter (PerkinElmer) was set with the excitation filter at 745, the emission filter at 770.

The fluorescence intensity for lung and brain was normalized to the respective organ weight.

A heat map has been set for the all the organ biodistribution. Specifically, the relative number of individual whole organs (y-axis) with a positive signal for each group (x-axis) was represented in a heat map. The accepted threshold for a positive signal was established at 50 arbitrary units (fluorescence intensity units of the spectrophotometer) as the lower level of sensitivity obtained by a dilution curve using cell homogenate labelled with DiR. Subtraction of the control signal was not applied to the heat map analysis. Color intensity, depicted on the bar at the right side of the figure ranges from 0 (0/4 samples with one positive replicate) to 1 (4/4 samples with one positive replicate), respectively. All organs with the signal below the 50 A.U. threshold were qualified as negative (0), and all organs with the signal above the threshold of 50 A.U. (regardless the maximum signal intensity) were qualified are positive.^1^

Lungs of rat pups were fixed in PFA 4% and embedded in OCT (Carl Roth), and slides were cut at 8 μm of thickness with cryostat. A solution (1% BSA + PBS) at RT for 2 hours was applied. To verify if the DiR-labelled EVs interacted with the lung epithelium, the slides were stained with EpCAM antibody (see Table 4) diluted in PBS + BSA 1% overnight at 4°C. Secondary antibody was applied for 1 hour at 37°C, and after washing, Fluoroshield with DAPI mounting medium (Sigma F6057) was used.

**Table 4.** Antibody list.

Antibodies	Cat number	Company
FITC Mouse, anti-Rat Granulocytes	554907	BD
APC-Cy7 Mouse, anti-rat CD45	561586	BD
BV605 Mouse anti-rat CD3	563949	BD
BV786 Mouse anti-rat CD172	744732	BD
APC anti-rat CD43	130-107-720	Miltenyi Biotech
PE mouse anti-rat CD68	130-123-757	Miltenyi Biotec
FITC mouse anti-αSMA	GTX72531	Genetex
Rabbit anti SFTPC	orb30333	Biorbyt
Mouse anti 8-oxo-dG	4354-MC-050	BIO-TECHNE
Goat anti GFAP	Ab53554	Abcam
Goat anti RAGE	AF1145	BIO-TECHNE
Rabbit anti EpCAM	GTX636759	Genetex
Rabbit anti Ki67	Ab15580	Abcam
Mouse anti CD68	MA5-13324	Invitrogen
Rabbit anti αSMA	ab5694	Abcam
Goat anti-Mouse IgG Secondary Antibody, Alexa Fluor 594	A11005	Life Technologies
Goat anti-Rabbit IgG Secondary Antibody, Alexa Fluor 594	A11006	Life Technologies
Chicken anti-Goat IgG (H + L) Cross-Adsorbed Secondary Antibody, Alexa Fluor 647	A21469	Life Technologies

### Hyperoxia Model

Following authorization (n°616/2016-PR released on June 17, 2016), at birth, Sprague Dawley outbred rat (Charles River Laboratory) pups were assigned randomly to the different experimental groups (10 pups/group); Normoxia + PBS group, Hyperoxia + PBS group and Hyperoxia + MSC-EVs group. Two experiments were performed for a total of 20 pups/group (60 pups in total). Overall survival was analyzed for all the experiments (Table 1).

**Table 1.** Summary of the 2 experiments. Number of pups per group are outside the brackets. Between brackets are the number of pups reaching the experimental endpoint.

Exp. *N*	Normoxia + PBS	Hyperoxia + PBS	Hyperoxia + MSC-EVs
#1	10 (10)	10 (7)	10 (10)
#2	10 (10)	10 (10)	10 (9)
#3	10 (10)	10 (9)	10 (10)
Survival (%)	100	86,6	96,6

The Normoxia group was maintained at room air (21% O_2_), and the Hyperoxia groups were placed in the OxyCycler at birth and exposed to 60% O_2_. The OxyCycler C42 (BioSpherix, OxyCycler model A84XOV, Redfield, NY) is a unique dual channel/dual gas controller for incubators and C-Chambers. Oxygen and carbon dioxide control is managed via independently programmable channels for optimum control^3^; https://biospherix.com/literature/). Every other day, the nursing dams are rotated in normoxia/hyperoxia conditions to prevent negative effects of hyperoxia on the breastfeeding process. Each experimental group included a balanced sample of rats across litters, to limit possible interfering effects caused by litter differences.

At 3 different time points (post-natal day (P), P3, P7, P10), the animals were anesthetized with 3% isofluorane for 5 minutes and the MSC-EVs, diluted with PBS 1× to the final concertation of 6.4E + 9 particles in a final volume of 50 µl, were IT injected in the pups of the hyperoxia-exposed group. The pups of the other control groups were injected with the same volume of PBS 1× as control. At P14, the pups were anesthetized with 3% isofluorane for 5 minutes and euthanized by severing the abdominal aorta.

### Organ and Cell Collection

#### Lung and Brain

After opening the thorax, the left bronchus was clamped, and the whole left lung collected by dissecting the left bronchus. For morphometric studies and immunofluorescence (IF), the right lung was insufflated with neutral buffered formalin (10%) at a pressure of 25 cm H_2_O and immersed in buffered formalin for 72 hours to allow for the complete fixation; brains were also processed in formalin. For cytofluorimetric analysis, the left lungs were collected (see Table 2) and placed in cold DMEM 4.5 g/L glucose + 1% P/S and processed. For molecular biology, and oxidative stress analysis, the left lungs and the brain were immediately snap-frozen in liquid nitrogen (see Table 2).

**Table 2.** Analysis of the organs: RL (Right Lung); LL (Left Lung) and Brain. Organs of surviving pups were processed for sample analysis.

	Organs									Process
	Normoxia + PBS			Hyperoxia + PBS			Hyperoxia + MSC-EVs			
	RL	LL	Brain	RL	LL	Brain	RL	LL	Brain	
Morphometry + IF	20	—	10	17		8	19	—	10	Formalin
qRT-PCR + carbonylation	—	10	10	—	9	9	—	9	9	Snap frozen
Flow cytometry	—	10	—	—	8	—	—	10	—	DMEM

#### Rat Macrophages

Macrophages were isolated from rat bone marrow by flushing femur and tibia of newborn (8 days) Sprague Dawley outbred rat (Charles River Laboratory) (approved ethical procedure from the Italian Law and local committee 862/2016 PR) with DMEM 1 g/L glucose, 10% FBS, 1% Penicillin/Streptomycin (P/S), and 1% L-Glutamine (Gibco). Recovered cells were plated in 24-well tissue culture Petri dish (Falcon) and stimulated with rat M-CSF (Peprotech) (10 ng/mL) for 7 days. Media was changed with different media for each experimental group: Ctrl group was treated with normal growth media; +TGFβ media was supplemented with recombinant TGFβ 10 ng/mL; TGFβ receptor inhibitor media was supplemented with recombinant TGFβ 10 ng/mL + 2,5 µM of TGFβ receptor I inhibitor SB431542 (Sigma S4317); MSC-EVs treated conditions were placed in media supplemented with recombinant TGFβ 10 ng/mL with the addition of 1E8 or 1E9 EVs/wells. Samples were analyzed before the stimulation (d0) and at different days after the stimulation (d2, d4, and d7). Samples were analyzed by immunofluorescence, Flow cytometry, and qRT-PCR.

### Cell Line

#### Human Alveolar Epithelial Cells

Human alveolar epithelial cells (CSC-C9223J, Creative Bioarray) were plated at passage 12 in 24-well tissue culture Petri dish (Falcon) with Supercult Alveolar Epithelial cell Medium (Creative Bioarray) (day0) at the density of 10 000 cells/well. At day 1, the medium was changed and supplemented with the stimuli: hydrogen peroxide 300 μM as prooxidative agent. At day 2, the medium was replaced and were tested 2 different concentrations of L-ascorbic acid (A4544-25G, Sigma) 50 and 100 μM versus 1E8 or 1E9 MSC-EVs/wells. At day 6, the damage was renewed, and at day 7, were added again the MSC-EVs and L-ascorbic acid. The cells were analyzed at day 6, day 8, and day 10 by immunofluorescence.

### Sample Analysis

In Table 2, number of lung and brain organs that underwent morphological analysis, immunofluorescence (IF), qRT-PCR, and cytofluorimetric analysis, are specified.

#### Molecular Biology, RNA Extraction, RT, and qRT-PCR

Total RNA was purified from lung samples (30-50 mg) using TRIzol reagent (Invitrogen, Thermo Fisher Scientific, Waltham, MA, USA #15596026) according to the manufacturer’s protocol. RNA concentration was assessed using Nanodrop 2000 (Thermo Fisher Scientific). High Capacity cDNA Reverse Transcription kit (Applied Biosystems) was used, and qRT-PCR was performed with 7500 Fast Real-Time PCR System, Applied biosystem. Housekeeping gene and primers sequence are listed in Table 3. 2^−ΔΔCt^ method for data quantification was used.

**Table 3.** Primer list.

Genes	Accession number	Amplicon size	Forward primer	Reverse primer
Tgf-β1	NM_021578.2	108pb	GTGGACCGCAACAACGCAAT Tm = 60°C	CAATGGGGGTTCTGGCACTG Tm: 60°C
Nrf2	NM_031789.2	124pb	GCCTTCCTCTGCTGCCATTA Tm = 60°C	ATTGAACTCCACCGTGCCTT Tm = 60°C
αSMA	NM_031004.2	188pb	CATCATGCGTCTGGACTTGG Tm = 59°C	CCAGGGAAGAAGAGGAGCA Tm = 59°C
B2micro globulin	NM_012512.2	98pb	ACACTGAATTCACACCCACCG Tm = 61°C	CATGTCTCGGTCCCAGGTGA Tm = 61°C
Sod2	NM_017051.2	157pb	CCCTGACCTGCCTTACGAC Tm = 60°C	AGTTGTAACATCTCCCTTGGC Tm = 60°C
Sftpc	U07796.1	172pb	TCTCATCGTGGTTGTGGTGG Tm = 60°C	AGCGATGGTGTCTGTGTGTT Tm = 60°C
Aqp5	NM_012779.2	135pb	GCTGAACAACAACACAACGC Tm = 59.09°C	CCAATGGATAAGGCTGGGGA Tm = 59.15°C
Col1a1	NM_053304.1	150pb	GCAACATGGAGACAGGTCAGA Tm = 60°C	TCGCTTCCATACTCGAACTGG Tm = 59,87°C
Col5a1	NM_134452.2	125pb	CTGAGGGAGCCAGAATCACT Tm = 60°C	CATTTGTACCACGCCCACAG Tm = 60°C

Abbreviations: *Tgf*β1: transforming growth factor 1; *αSMA*: Alpha Smooth muscle actin; *Nrf2*: nuclear factor erythroid 2-related factor 2; *Sod2*: superoxide dismutase 2; *Sftpc*: surfactant protein C; *Aqp5*: aquaporin 5; *Col1a1*: collagen 1a1; *Col5a1*: collagen 5a1.

#### Protein Carbonylation

50 mg of sample were homogenized in buffer (Tris-HCl 20 mM buffer, pH 7.6, EDTA 1 mM, DTT 1 mM, sucrose 0.5 M, and KCl 0.15 M), for 10 mg of samples were used 50 μL of homogenization buffer. The protein carbonylation assay was performed according to the manufacturer instruction of the Protein Carbonyl content assay kit (Sigma). The results were normalized using total protein concentration obtained using Pierce BCA Protein assay kit (Thermo Fisher Scientific).

#### Flow Cytometry

About 140 mg of the left lung were digested according to the protocol previously published,^19^ and briefly, organs were cut in small pieces with scissors and incubated for 45 minutes at 37°C on horizontal shaking in digestion medium. To each well, 1 mL of Digestion Medium (HBSS + 5% FBS + 10mM HEPES), supplemented with 1.5 mg/mL collagenase II (Thermo Fisher Scientific) and 0.4 mg/mL DNase I (Roche) was added. Organs were incubated for 45 minutes at 37°C on horizontal shaking. An equal volume of enzyme containing Digestion Medium was supplemented to each well and incubated again for 45 minutes at 37°C on horizontal shaking. Digested tissues were collected, and the cell suspension was filtered onto a 100 µm cell strainer (Falcon #352360) and centrifuged for 10 minutes at 300*g*. Supernatant was removed, and pellet was resuspended in ACK lysis buffer (Lonza) and incubated for 5 minutes at room temperature. ACK was blocked with PBS, collected by pipetting and the cell suspension was passed onto a 100 µm cell strainer. Cells were centrifuged for 10 minutes at 300*g*. Supernatant was removed, and pelleted cells were resuspended in PBS and split in conditions for FACS analysis: lymphocytes (CD45+ CD3+); resident macrophages (CD45+, CD43low, His48med, CD172+); monocytes/macrophages (CD45+, CD43hi, His48med-low); and proinflammatory macrophages (CD45+, CD43lo, His48hi). Staining was performed in FACS buffer (PBS+ 2% FBS) for 30 minutes at 4°C employing antibodies listed in Table 4 diluted 1:100. Samples were washed in FACS buffer and centrifuged for 2 minutes at 2000*g*. Samples were read at BD FACSCelesta Flow Cytometer. Sample were normalized on normoxia.

Cells were detached using Accutase (Thermo Fisher), separated in different tubes for each condition, treated with Fix and Perm (Thermo Fisher) stained with antibodies coupled with fluorophores as in Table 4. Cells were analyzed using Cytoflex (Beckman Coulter), and results are expressed as percentage of cells positive for each fluorophore.

#### Immunofluorescence (IF)

Paraffin-embedded samples were rehydrated and dewaxed by placing the slides in Xilene and then ethanol solutions (100%/100%/95%/80%) and distilled water. Slides heated at 90°C in Citrate buffer solution (Trisodium Citrate 10mM pH 6.03 + 0.05% Tween-20) for 40 minutes. Slides were put in a solution of Glycine 0.1M in PBS for 1 hour. A solution (1% BSA + PBS + 0,1% Triton X-100) at RT for 2 hours was applied. Primary antibodies listed in Table 4 diluted in PBS + BSA 1% overnight at 4°C were used. Secondary antibodies were applied for 1 hour at 37°C, and after washing, Fluoroshield with DAPI mounting medium (Sigma F6057) was used.

Cells were fixed with a 4% PFA solution for 10 minutes, blocked with a solution of 10% horse serum in PBS and stained with primary and secondary antibodies accordingly to Table 4. DAPI was used to stain cells nuclei. Ten pictures for each sample with a 20× magnitude using Leica B5000 inverted microscope were taken. Positive cells were counted, and the results were expressed as the percentage of positive cells over the total cells.

To quantify the amount of αSMA in lung macrophages, lungs were stained with primary antibodies for the macrophage marker CD68 and αSMA. Pictures of single CD68 positive cells were taken using Zeiss LSM 800 Airyscan confocal microscopy. In each picture, the fluorescence of αSMA was calculated using Fiji software, and results were normalized on the Hyperoxic + PBS condition.

#### Molecular Biology

##### RNA Extraction, RT, and qRT-PCR

Total RNA was purified from cells using TRIzol reagent (Invitrogen, Thermo Fisher Scientific) according to the manufacturer’s protocol. RNA concentration was assessed using Nanodrop 2000 (Thermo Fisher Scientific). HighCapacity cDNA Reverse Transcription kit (Applied Biosystems) was used, and qRT-PCR was performed with 7500 Fast Real-Time PCR System, Applied biosystem. Housekeeping gene and primers sequence are listed on Table 3. 2^−ΔCt^ method for data quantification was used.

### Histology

Paraffin-embedded samples were rehydrated and dewaxed by placing the slides first in xylene and then ethanol solutions (100%/100%/95%/80%) and distilled water.

#### Sirius Red

First, hematoxylin staining was performed for nuclei using Gills Hematoxylin solution nr.3 (Bio Optica) for 8 minutes. Slides were washed in running water and then staining with Sirius red solution (Sigma) was performed with incubation of the slides in the staining solution for 40 minutes. After incubation, excess of Sirius red was removed with a paper, and slides were washed 2 times in acidified water. Slides were then dehydrated with 100% ethanol and xylene and then mounted with Eukitt mounting medium (Fluka). Ten random pictures were taken at 10× magnitude using Olympus IX71 microscope (Olympus), and quantification of collagen was performed with Fiji software, using the plugin color deconvolution for Fast Red Fast Blu DAB, using this parameter was possible to isolate collagen staining, that was quantified using Fiji’s Threshold software. Since threshold provide us the percentage of area stained in comparison with the dimension of the picture, results were expressed as Collagen I deposition/field (%).

#### Azan-Mallory

Samples were stained using Azan trichrome kit (Bio Optica) following manufacturer instructions. Eukitt mounting medium (Fluka) was used to mount the slide. 20× pictures were taken.

#### Alcian Blue

Samples were stained for glycosaminoglycans using Alcian blue pH 2,5 (Bio Optica) following manufacturer instructions. Eukitt mounting medium (Fluka) was used. 20× magnitude images using Olympus IX71 microscope were processed using Fiji software (plugin Color deconvolution for Alcian blue). Normal threshold of the lung tissue stained in blue has been performed. This process will express the results as percentage of pixels positive for Alcian blue staining over the total pixels in the picture.

#### Movat Pentachrome Stain

Samples were stained using the Movat pentachromatic stain kit (Diapath, Bergamo, Italy) and following the manufacturer’s protocol. Briefly, after section rehydration, dewaxing, and incubation in graded ethanol series, samples were washed with running water for 5 minutes, and incubation with the solutions present in the kit was performed. After the procedure, slides were finally mounted with Eukitt mounting medium (Fluka).^20^

### TEM

Lung tissue samples (about 1-2 mm^3^) were fixed in 2.5% glutaraldehyde plus 2% paraformaldehyde in 0.1 M sodium cacodylate buffer pH 7.4 O.N. at 4°C, subsequently postfixed in osmium tetroxide 1% in 0.1 M sodium cacodylate buffer for 2 hours at 4°C and embedded in an Epon-Araldite resin mixture. The tissue has been processed in order to analyze the central part of the lungs that is comprehensive of epithelial cells. Ultrathin sections (60-70 nm) were obtained with a Leica Ultracut EM UC7 ultramicrotome, counterstained with uranyl acetate and lead citrate, and viewed with a Tecnai G^2^ (FEI) transmission electron microscope operating at 100 kV. Images were captured with Veleta (Olympus Soft Imaging System corporation, Tokyo, Japan) camera, and processed for mitochondria and lamellar bodies shape parameters detection using Fiji software.

## Results

### MSC-EVs Biodistribution and Alveolarization Analysis

CryoTEM showed the MSC-EVs morphology and cytofluorimetric results confirmed expression of classical tetraspanins CD9, CD63, CD81 in the EVs (Supplementary Fig. S2A). Finally, we confirmed that the DiR staining on EVs does not affect the size, or the number of particles measured by NTA (Supplementary Fig. S2B).

**Figure 1.**
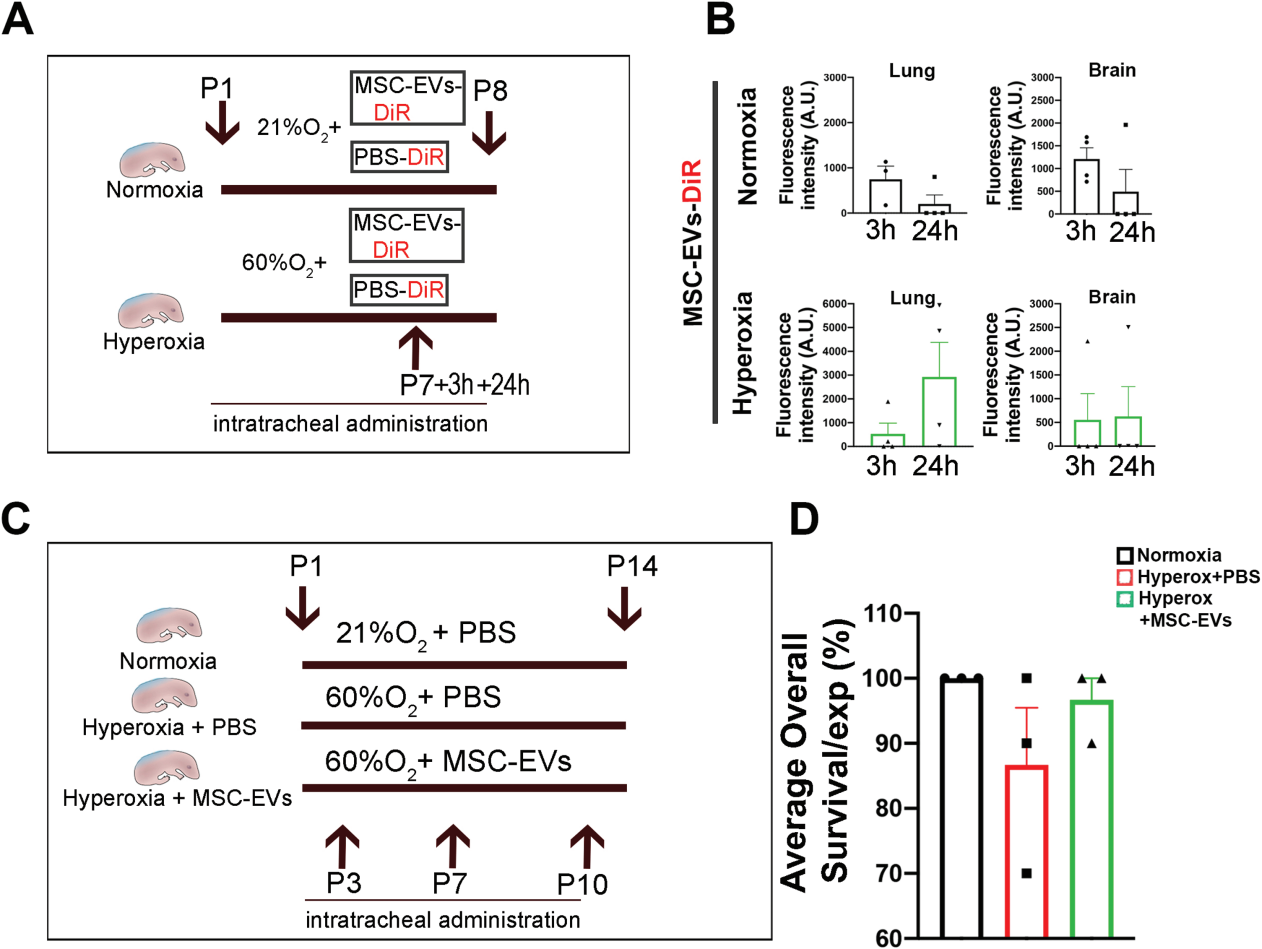
Lung damage evaluation. (**A**) Experimental plan of the biodistribution study. (**B**). MSC-EVs-DiR fluorescence detection in lung and brain in normoxia (upper lane) and in hyperoxia (lower lane) groups. (**C**) Experimental plan of the efficacy study. (**D**) Overall survival of the different groups. *N* of experiments = 3. *N*° of pup rat/group: 30.

Biodistribution analysis showed that labelled clinical grade MSC-EVs intratracheally administrated were found in lung and brain at 3 and 24 hours postinjection (Fig. 1A, 1B).

In the other organs, MSC-EVs were distributed in an evenly manner (Supplementary Fig. S1).

When looking more closely at the biodistribution of labelled EVs in the lungs, we were able to detect spot area in which the epithelial marker EpCAM overlapped with DiR-EV signal, confirming a specific delivery of MSC-EVs to the epithelial cells in the lungs (Supplementary Fig. S3A). Furthermore, in line with previous published data obtained in our group,^3^ we confirmed that MSC-EVs exert a protective role against hyperoxia-induced lethality in rat pups (Fig. 1C, 1D).

Morphometric analysis of alveolarization was also consistent with previous work^3^ (Supplementary Table S1). The hyperoxia PBS-treated group showed no significant changes in terms of lung volume (V_lung_) and volume fractions of septa with respect to the normoxia-exposed animals. On the other hand, with respect to the latter group, the former one showed lower volume fraction of airspaces, lower total surface area of alveolar air spaces, higher mean intercept length, higher thickness of septa, lower total number of alveoli, and higher mean alveolar volume. Upon hyperoxia, compared with the hyperoxia PBS-treated group, the MSC-EVs-treated group showed significant increases in total surface area of alveolar air spaces and in total number of alveoli and significant reductions in thickness of alveolar septa and mean alveolar volume. Thus, EVs-treatment confirmed its efficacy in preventing hyperoxia-induced changes in alveolarization, partially restoring a normal lung phenotype.

### MSC-EVs Protect Lung Epithelial Cells From Hyperoxic Damage and Reach the Brain

**Figure 2.**
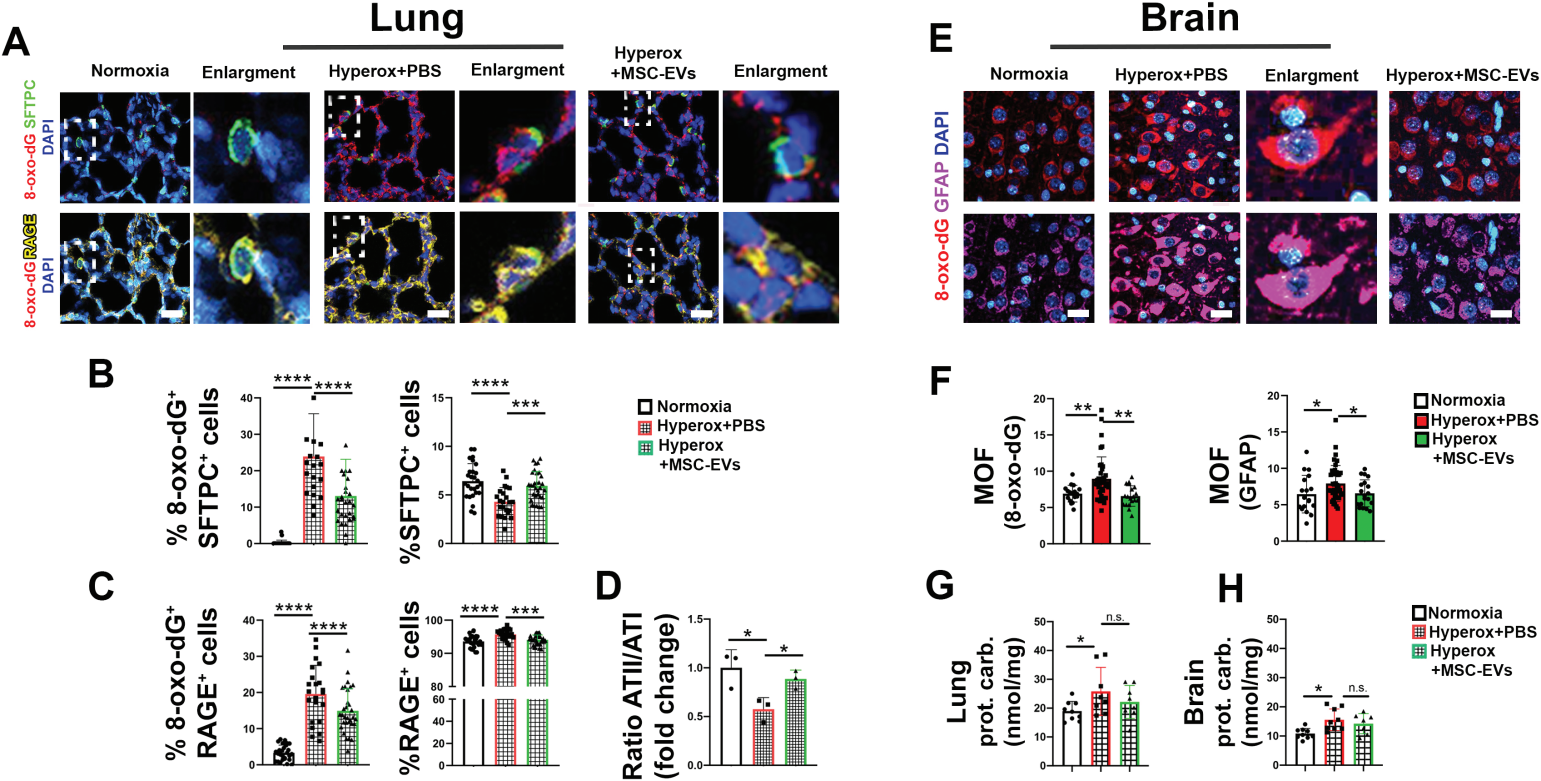
Hyperoxic damage and epithelial compartment. (**A**) SFPTC (surfactant protein C) IF for ATII cells, RAGE (Receptor for Advanced Glycation Endproducts) for ATI cells in the 3 different groups. Scale bar = 25 μm. (**B**) 8-oxo-dG and SFTPC positive cell quantification. (**C**) 8-oxo-dG and RAGE positive cell quantification. (**D**) Ratio in fold change of ATII/ATI cells. (**E**) 8-oxo-dG and GFAP IF. Scale bar = 25 μm. (**F**) 8-oxo-dG and GFAP mean of fluorescence (MOF) quantification. (**G**, **H**) Lung and brain protein carbonylation. *P < .05; **P < 01; ***P < .001; ****P < .0001.

We further investigated the antioxidant effect of the nanoparticles in the lung by quantifying the DNA oxidative stress biomarker 8-oxo-dG. Overall, upon hyperoxia, this antibody gave a positive signal in both ATI and ATII cells while in the lungs of animals left in normoxia, no 8-oxo-dG staining was detected. The signal was the highest in the hyperoxic + PBS injected pups and treatment with MSC-EVs strongly reduced the detected 8-oxo-dG levels, once again suggesting a protective role of these nanoparticles. Only a small percentage of ATII cells are still present, and probably they are stimulated to differentiate into ATI in order to replace the damaged cells (Fig. 2A-2C).

Interestingly, the ratio between ATI and ATII cells upon hyperoxia is affected (Fig. 2D). ATI alveolar epithelial cells deputed to gas exchange that derived from the maturation of ATII and express RAGE (Receptor for Advanced Glycation Endproducts) are significantly increased compared to pups left in normoxia. In reverse, ATII cells (shown by SFTPC staining) are significantly decreased in this condition. Of most interest, treatment with MSC-EVs restores a normoxia-like ratio between these 2 cell types (Fig. 2A-2D). This confirms that in the hyperoxia condition, the epithelium of the lung is almost solely composed of damaged ATI cells. Of note, to complement these observations we investigated the genes expressed by ATI and ATII cells, namely *Aquaporin5* (*Aqp5*) and *Sftpc*. Interestingly, expression levels of *Aqp5* for ATI cells and *Sftpc* for ATII cells did not show the same correlation of the protein expression (RAGE and SFTPC) among the groups (Supplementary Fig. S3B). Yet, it is important to note that this discrepancy could be due to the technical approach because while the microscopy technique enables to investigate the expression per cell, total qRT-PCR analysis relies on a homogenate of cells, therefore, masking single-cell expression.

The lung histological analysis highlighted differences among the groups (as already proved^3^). In fact, the exposure to hyperoxia caused impaired alveolar development in the PBS-treated group, with fewer and larger distal airspaces, a reduction of secondary alveolar crests and evident interstitial thickening with respect to the lungs of pups maintained in normoxic and MSC-EVs conditions.

Regarding the brain, while the histological staining did not evidence substantial differences among the 3 groups (Supplementary Fig. S4), we found peculiar results with immunofluorescence. High levels of 8-oxo-dG in the normoxia condition (Fig. 2D, 2E) were detected. This is a known characteristic of the young rats,^21^ probably due to naturally high brain metabolism and the high basal production of reactive oxidative species (ROS), consequence of the rapid changes during the brain development in this mammal. In normoxia, 8-oxo-dG was present only in glial fibrillary acidic protein (GFAP) positive cells such as astrocytes. We found an increase of DNA oxidation in the damaged group, shown by the intensity of the signals, and again with the MSC-EVs we observed a significative reduction of 8-oxo-dG signal. The mean fluorescence intensity of GFAP followed the same trend of 8-oxo-dG, meaning that hyperoxia-derived ROS activated astrocytes and treatment with MSC-EVs help to restore and maintain their homeostasis.

As an additional mechanism induced by hyperoxia and redox stress, we considered protein carbonylation as a new aspect of damage that mimics BPD. Both in lung and in brain carbonylation (measured by total carbonylation protein on tissue homogenate) appeared significatively higher in hyperoxia respect to normoxia pups. Once again, MSC-EVs treatment tend to reduce the level of carbonylated proteins, yet without reaching significance (Fig. 2F, 2G). This is in line with the fact that protein carbonylation is an irreversible process. Nevertheless, MSC-EVs demonstrated again to exert protection against damage progression in BPD-like condition.

**Figure 3.**
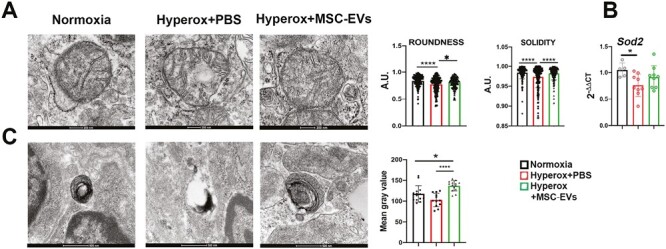
Ultrastructure analysis of the lung. (**A**) TEM of lung mitochondria. Scale bar = 200 nm. Quantification of shape parameters of mitochondria (roundness and solidity). Counted 200 mitochondria per group. Student’s *t*-test *P < .05, ****P < .0001. (**B**) *Sod2* enzymatic evaluation. (**C**) Lamellar body and evaluation of the structures present in the 3 different groups. Scale bar = 500 nm. Student’s *t*-test (Statistical analysis was made comparing 2 groups.) *P < .05, ****P < .0001.

To further investigate the response of lung epithelial cells to hyperoxia, we also investigated mitochondrial homeostasis. Mitochondria analysis showed that hyperoxia induces a disorganization of the mitochondrial that appear less round (roundness in the graph) coupled with an overall reduction of electron density (solidity in the graph) in the mitochondria, suggesting a mitochondrial stress (Fig. 3A). Therefore, the roundness and the solidity of mitochondria retrieved from pups grown in hyperoxia were significantly lower in respect to the MSC-EVs treated or normoxia-treated pups (Fig. 3A). These results were further confirmed by analysis of lamellar bodies that were less dense upon hyperoxia and more dense when treated with MSC-EVs (Fig. 3C). Finally, to complement these data, we investigated the mRNA expression level of the specific mitochondrial enzyme superoxide dismutase 2 (*Sod2*).^22,23^ We observed that *Sod2* mRNA levels were affected upon hyperoxia, probably because the mitochondria in this condition are strongly damaged. MSC-EVs were able to partially restore the *Sod2* mRNA expression. This leads us to hypothesise that the enzymatic activity of *Sod2* is a downstream effect, plays a role in the protective cascade induced by MSC-EVs to counteract hyperoxic stress (Fig. 3B).

As described earlier, the epithelial lung compartment is paramount for lung function. Glycosaminoglycans stimulate alveolar type II cells (ATII) cell proliferation and surfactant (SFTPC) is the main protein secreted by healthy alveolar ATII cells. These cells are deputed to epithelial regeneration when the alveolar compartment is damaged.

We evaluated the non-sulphated glycosaminoglycans (GAGs), such as hyaluronic acid, using Alcian blue staining and Movat staining, and we showed a decreased in glycosaminoglycans (Supplementary Fig. S5A-5D) in the pups that endure hyperoxia. In parallel, also SFTPC diminished in hyperoxia-treated pups’ lungs, while MSC-EVs treatment induced restoration of GAGs and SFTPC expression to levels higher than the normoxia control (Fig. 2B) group.

Histopathological analysis confirmed the findings of previous study.^3^ Lung sections showed signs of impaired alveolar development in the PBS animals exposed to hyperoxia, with distal air spaces which were fewer in number and larger in diameter, with reduced septation and patchy areas of marked interstitial thickening. EVs-treatment clearly reduced these histopathological features (Supplementary Table S2).

Taken together, these results underlined that MSC-EVs exert a protective effect also in the lung epithelial compartment.

### The Fibrosis Process Is Counteracted by MSC-EVs

**Figure 4.**
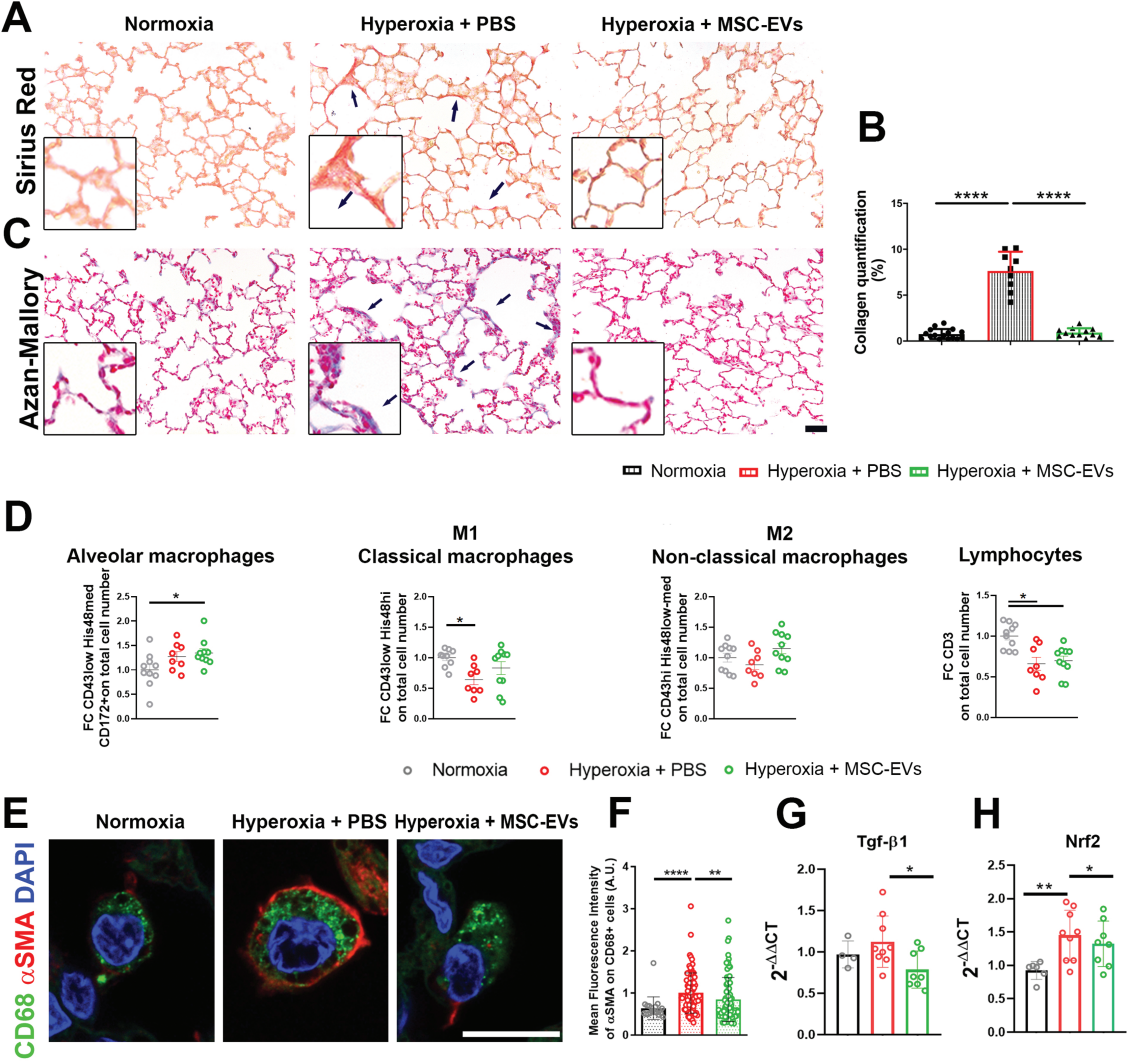
The macrophage compartment in the lung. (**A**) Left: Sirius red staining to evaluate collagen deposition. Scale bar = 25 μm. (**B**) Upper row: Collagen quantification in the different groups. Lower row: qRT-PCR of *Collagen 1A1* and *5A1*. (**C**) Azan-Mallory staining to identify connective tissue and collagen deposition. (**D**) Fresh cytofluorimetric analysis of cells retrieved from the lung. Lymphocytes and different macrophages populations have been analyzed. (**E**) Immunofluorescence for CD68 (green) and αSMA (red) in lung paraffin embedded. Scale bar = 125 μm. (**F**) Mean fluorescence intensity of αSMA on CD68 in the different groups. (**G**) Tgfβ1 qRT-PCR in lungs. (**H**) *Nrf2* qRT-PCR in lungs. *P<.05; **P < .01; ****P < .0001.

The lung epithelial damage is often associated with the initiation of fibrotic events.^24^ Thus, we wondered whether a collagen deposition was detectable in our model as result of the hyperoxia-driven epithelial insult. While Movat staining did not detect substantial difference in collagen presence in the different groups (Supplementary Fig. S5D), not surprisingly, Sirius red and Azan-Mallory immunohistochemistry proved a higher lung fibrosis in hyperoxic pups in comparison with the normoxic group. However, the gene expression of *Collagen 1A1* and *5A1* was not comparable with the immunohistochemical detection of the collagen protein (Fig. 4B). Importantly, the MSC-EVs treatment significantly prevented the hyperoxia-induced collagen deposition (Fig. 4A-4C).

To investigate the cell compartment involved in this altered process, immune cells of freshly isolated lungs were analyzed by flow cytometry. Notably, MSC-EVs treatment increased the percentage of resident alveolar macrophages (CD43low−His48high−CD172+) compared to the untreated rats. In addition, MSC-EVs treatment showed a trend in rescuing the percentage of classical macrophages (CD43low−His48high), which were reduced in the hyperoxic condition. Concomitantly, non-classical macrophages, identified as CD43high−His48low cells, were not affected neither in hyperoxic conditions or in MSC-EVs treated rats. To note, uniquely the macrophage compartment seems to be affected by the MSC-EVs treatment. Indeed, no differences between hyperoxia+ PBS and hy peroxia+ MSC-EVs groups were reported when the lymphocytic compartment (CD3+ cells) was considered (Fig. 4D).

To evaluate the ability of macrophages to concur to the myofibroblast generation, the main cell type responsible for collagen production, a marker of macrophages (CD68), and a marker of myofibroblasts (αSMA) were considered by immunofluorescence in lungs of treated and untreated animals. Surprisingly, as shown in Fig. 4E and 4F, we noted a coexpression of these markers especially in hyperoxic pups suggesting a macrophage switch toward myofibroblasts. Furthermore, the MSC-EVs treatment significantly blocked this CD68+/αSMA+ macrophages lung accumulation (Fig. 4E and 4F) thus highlighting a putative pathway of action to be deeply investigated. This finding was also supported by the increased Tgfβ1 gene synthesis in hyperoxia condition as well as for *Nrf2* gene (Fig. 4G and 4H) also rescue upon MSC-EVs treatment.

### MSC-EVs Interfere With the Switch of Macrophages Toward Myofibroblast to Prevent Fibrosis and Oxidative Stress

**Figure 5.**
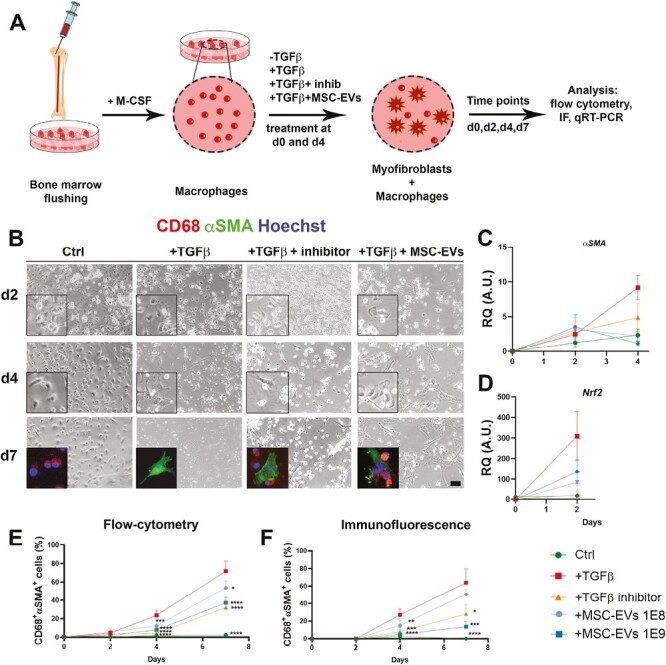
MSC-EVs interaction with myofibroblasts. (**A**) Experimental plan to stimulate primary rat macrophages toward myofibroblasts. (**B**) Phase-contrast images and IF of rat macrophages after TGFβ stimulation at different time points. Scale bar = 25 μm. (**C**) qRT-PCR for *αSMA* gene. (**D**) qRT-PCR for *Nrf2* gene. (**E**) Cytofluorimetric analysis of αSMA and CD68 coexpression at 2,4, 7 days post-TGFβ stimulation. (**F**) Immunofluorescence quantification of αSMA and CD68 coexpression at different time points. *P < .05; **P < .01; ***P < .001; ****P < .0001.

In order to prove the influence of MSC-EVs in switching macrophages toward myofibroblasts, we adapted a model of fibrosis from primary macrophages stimulated with TGFβ^25^ (Fig. 5A).

**Figure 6.**
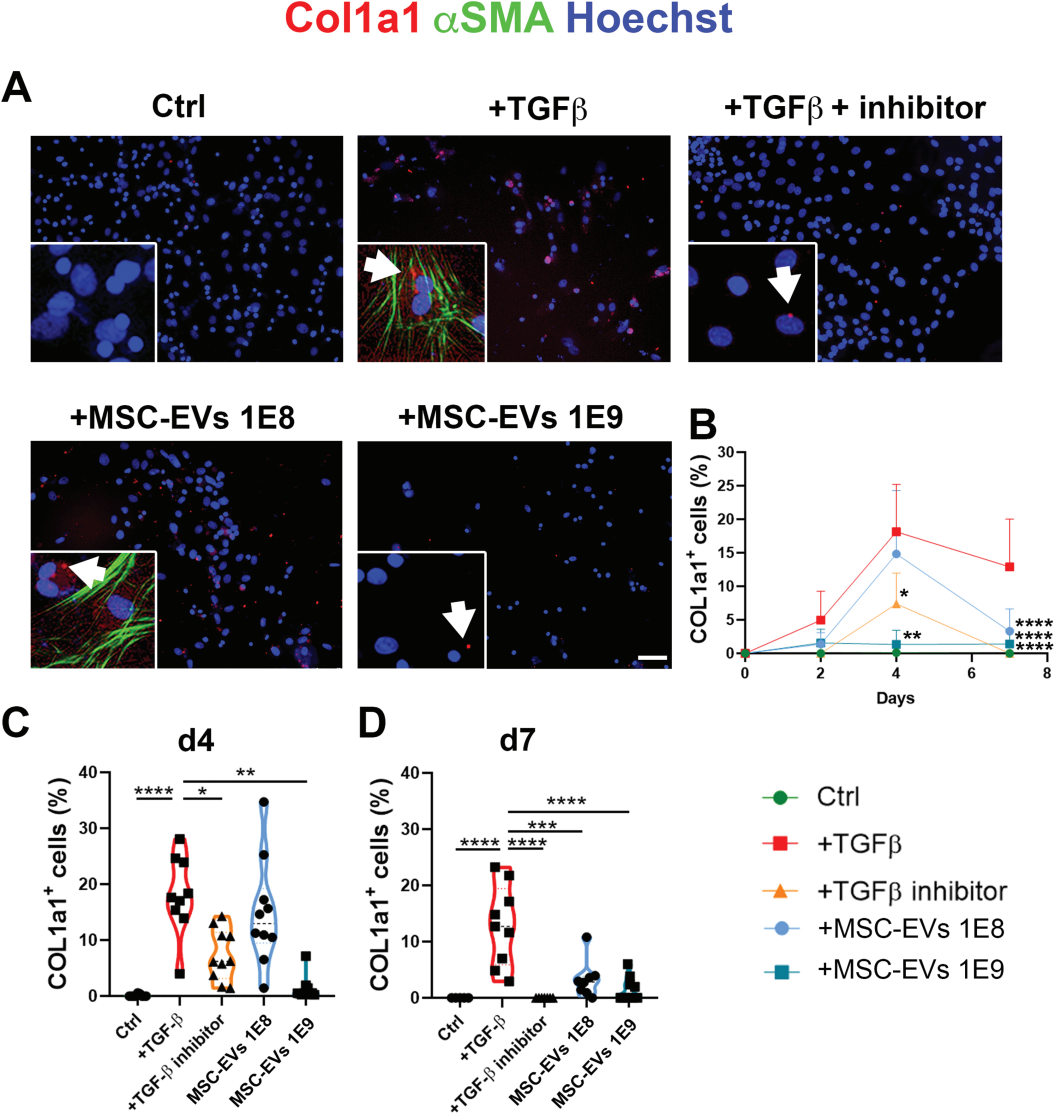
Collagen is secreted mainly by myofibroblasts. (**A**) Collagen I and α-SMA IF in the different analyzed conditions. Scale bar = 25 μm. (**B**) Collagen I a1 quantification at different time points. (**C**). Immunofluorescence quantification of Collagen I a1 expression at day 4. (**D**) Immunofluorescence quantification of Collagen I a1 expression at day 7. *P < .05; **P < .01; ***P < .001; ****P < .0001.

At first, both by immunofluorescence quantification and cytofluorimetric analysis, we demonstrated that after 2, 4, and 7 days TGFβ stimulation, αSMA expression increased in a time dependent manner and decreased according to the higher dose of MSC-EVs, more effective (Fig. 5B, 5E, 5F). Second, αSMA gene expression decreased after MSC-EVs administration while *Nrf2* was particularly expressed in cells with macrophage characteristics (Fig. 5C and 5D). Finally, we evaluated the collagen deposit in this in vitro model. With a double staining, we investigated αSMA positive cells, shown in green, and the presence of Col I (shown in red). Once again, MSC-EVs were able to decrease both stress markers of myofibrobastic fate (Fig. 6A). Quantification of COL1a1-positive cell over time (Fig. 6B) corroborated these observations. Of most interest, while only the higher concentration of MSC-EVs was able to significantly decrease collagen deposit at day 4 postinduction, at day 7, both the lower and the higher concentration of nanoparticles decreased collagen to a control-like level (Fig. 6C and 6D). The latter suggest not only a protective role of MSC-EVs but also a potential therapeutic effect, restoring the macrophage phenotype and response after being in contact with a stress. These results confirmed the influence of MSC-EVs on macrophage compartment, myofibroblast switching, fibrosis, and oxidative stress protection.

### MSC-EVs Protect the Lung Epithelium From DNA Oxidation

**Figure 7.**
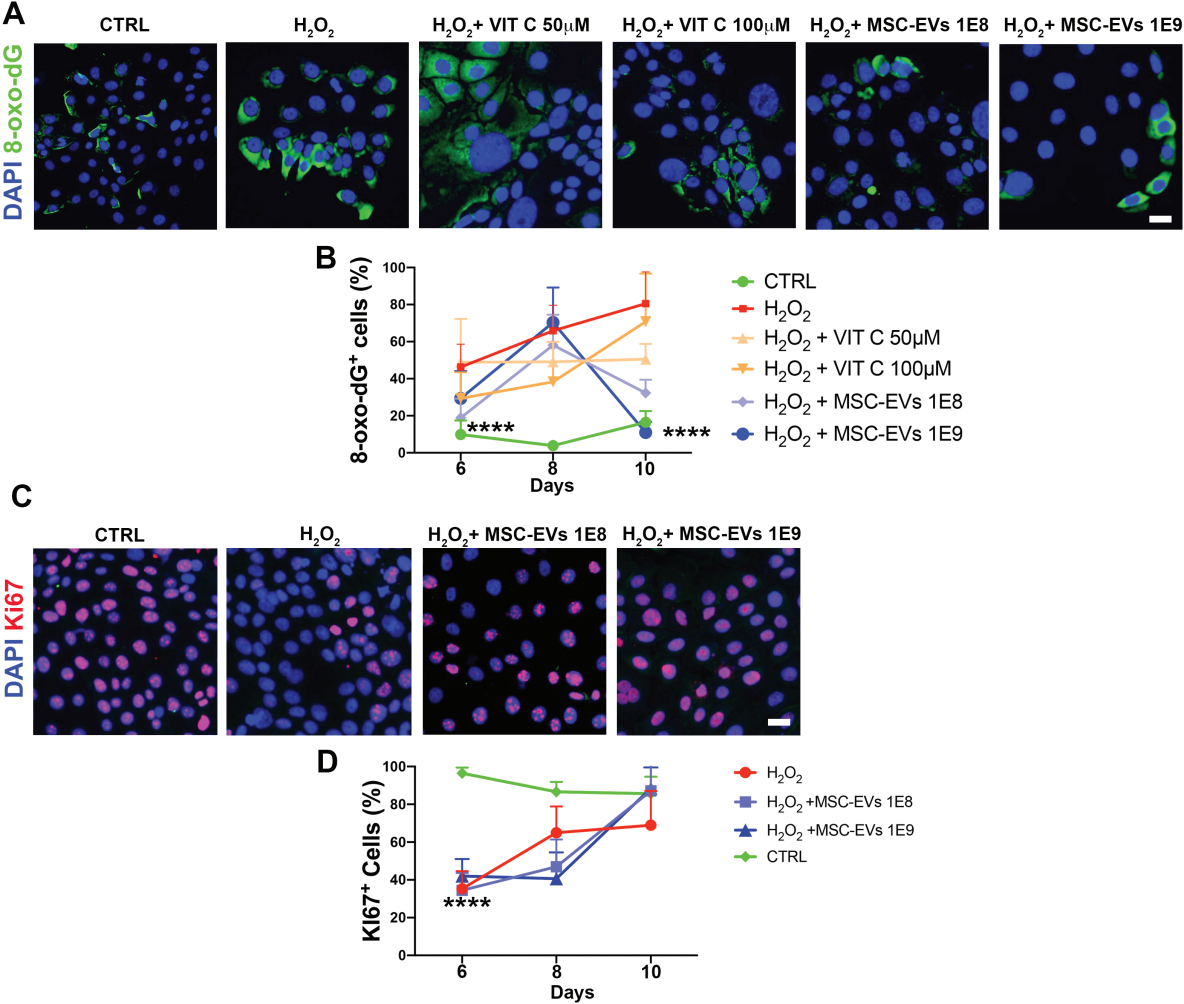
MSC-EVs protect from DNA oxidation. (**A**) Representative images of IF of the oxidative marker 8-oxo-dG in lung epithelial cells before and after H_2_O_2_ damage and after treatment with MSC-EVs and Vitamin C. (**B**) Percentage of cells positive for 8-oxo-dG at different time points. For detail statistical see Supplementary Table S3. (**C**) Representative images of IF of Ki67 before and after H_2_O_2_ damage and after treatment with MSC-EVs. (**D**). Percentage of cells positive for Ki67 at different time points. Scale bar = 75 μm. ****P < .0001.

To answer whether MSC-EVs exert their protection on epithelial cells, in vitro lung epithelial cells were damaged with H_2_O_2._ The H_2_O_2_ significantly inhibited the proliferation of the cells proving the attained oxidative injury (Fig. 7C and 7D). In the damaged cells, MSC-EVs and the antioxidant Vitamin C were administered. The oxidative marker 8-oxo-dG was analyzed at different time points and reveals to be directly proportional with the time of damage. Indeed, at day 10, a significant reduction of DNA oxidation was observed with both Vitamin C and the MSC-EVs. However, MSC-EVs demonstrated to scavenge better the ROS action than Vitamin C (Fig. 7A and 7B) and also in a dose-dependent manner. Of note, the mechanism of 8-oxo-dG repair induces the blockage of the cell cycle and the proliferation,^26^ as shown by our results at day 8 (graph in Fig. 7D). At day 10, the oxidation was indeed relapsed in MSC-EVs treated cells (graph in Fig. 7B). These results highlighted that the MSC-EVs can directly protect the lung epithelium against oxidative stress.

## Discussion

Oxidative stress has a central role in the development of BPD. In premature newborns, oxidative stress can result both from prolonged hyperoxia exposure during artificial ventilation and from the lung inflammatory response. The overproduction of ROS, mainly in mitochondria, results in a direct damage of proteins, lipids and nucleic acids, and in cell death through a variety of pathways, leading to diffuse alveolar injury.^27^ In particular, oxidative stress induces excessive apoptosis of alveolar epithelial type-II (stem) cells (AECII) and inhibits their proliferation, which is crucial for structural and functional repair of alveolar epithelial injury.^28^ In addition, excess of ROS production enhances signalling in promoting fibrosis via the TGFβ2/SMAD2 pathway.^29^ Finally, oxidative injury is found in other organs than the lung, including the brain and the eyes.^30,31^

In our work, we had the opportunity to use human clinical grade MSC-EVs in a xenogenic model. Following the MISEV guideline,^32^ there is no standard method of EVs separation and concentration, and several different techniques are equally acceptable, including (tangential flow filtration [TFF]). These EVs were newly produced in respect to our previous work in which also clinical-grade MSC-EVs have been used,^3^ and importantly, we excluded rejection effects. First, we demonstrated that MSC-EVs reached the damaged lung and passed through the brain barrier, with no accumulation effect over time. Second, we underlined the protective effects of MSC-EVs against oxidative stress injury that has been shown already in several organs, including kidneys, colon, heart, brain, and liver.^1,33-36^ However, the mode of action remains to be elucidated. From the literature, we understand that in injured colon, the therapeutic effect of MSC-EVs associated with suppression of oxidative perturbations was manifested by a decrease in the activity of myeloperoxidase and malondialdehyde, as well as with an increase in superoxide dismutase and glutathione.^1^ In the heart following ischemia/reperfusion injury, the hypothesized mode of action was by replenishing glycolytic enzymes to increase ATP production and supplementing additional protein components of the cellular antioxidant system, including peroxiredoxins and glutathione S-transferases.^33^ Finally, in clinically relevant models of ARDS, MSC-EVs containing mitochondria restored epithelial barrier integrity and normal levels of oxidative phosphorylation while an MSC-EVs preparation which did not contain mitochondria, was not effective.^37^ Our data show that the lung morphological improvement in MSC-EVs-treated animals is associated with a decrease in markers of oxidative stress, such as 8-oxo-dG. 8-oxo-dG is an established marker of DNA damage elicited by ROS. Guanine is the DNA base most susceptible to oxidative damage by ROS, leading to the production of 8-oxo-dG. The cell can recognize the oxidized nucleotide and remove it by the BER repair system.^38^ Failure to repair this damage leads to transversion GC → AT, altering the genetic information. In our model, MSC-EVs administration strongly reduced the concentration of 8-oxo-dG both in the lung and in the brain, demonstrating an important protective effect of these nanoparticles against DNA oxidation. The oxidative damage reached both ATII and ATI cells of the lung, and it may be foreseen that, although in small number, ATII cells differentiate into ATI.^14^ We confirmed the antioxidative property of MSC-EVs also in vitro when after the oxidative damage of H_2_O_2_, we used the antioxidant Vitamin C that was able to decrease the percentage of damaged cells, although in a less specific manner in respect to the MSC-EVs. H_2_O_2_ is a ROS that resembles the oxidative damage produced by hyperoxia in our in vivo model.^39^ Furthermore, we found that the cells that are susceptible to DNA oxidation are astrocytes in rat brain. Astrocytes are glial cells that are involved in the ionic homeostasis, in the maintenance of the brain-blood barrier (BBB) and are deputed to trophic and metabolomic support of neurons. Neurons do not produce significant antioxidant defences and this function is exploited by astrocytes.^40^ Since astrocytes exert this kind of protection, they should be the most reactive cells during modifications in the brain environment. Our results suggest that astrocytes are the first cell type that reacts to oxidative stress and are activated by ROS production inducing their activation and GFAP overexpression. MSC-EVs can protect the neuronal compartment reducing the oxidation and the expression of GFAP. The protein carbonylation has also been evaluated. This process leads to the protein oxidation, producing irreversible unfolding or alteration of protein structure.^41^ Carbonyl concentration was increased following exposure to hyperoxia, and this increase was blunted, albeit not significantly, in the EVs-treated group.

The improvement at the level of mitochondria is of particular interest, since these organelles are major producers of ROS, as reported above. This improvement was associated with decreased collagen deposition, suggesting that MSC-EVs could interfere with pathological processes linking oxidative stress with fibrosis. Clearly, this hypothesis requires further investigation. Of note, the increased expression of surfactant protein C reflects improved proliferation and/or function of AECII, a finding that could at least partially explain the morphological recovery of MSC-EVs-treated lungs from hyperoxic injury,^28^ but more with the appearance of the lamellar bodies at TEM microscope. In fact, we qualitatively compared the ultrastructure of the lamellar bodies (LB), secretory organelles located in ATII cells that store surfactant. From the literature, it is known that the structure of the LB can change according to the surfactant production influenced by particular genetic disorders and by stressful conditions in the lung environment.^42,43^ Our evaluation on LB showed that the ultrastructure of LB in hyperoxia-PBS group is less electrodense than the other 2 conditions. In particular, the appearance of the LB of hyperoxia-MSC-EVs is comparable to the normoxia LB. One possible explanation can be found in the switch/maturation of ATII cells toward ATI where the production of surfactant is absent.^14^ In addition, also ROS stimulate inflammation signal around mitochondria inducing the expansion of the cellular damage.^44^

Importantly, we report that MSC-EVs administration is associated with a significantly decreased oxidative brain injury in hyperoxia-exposed animals. This finding confirms recent observations that MSC-EVs administration can improve brain injury associated with hyperoxia in a BPD model,^45,46^ and that such a beneficial effect on organs other than the lung can be achieved with intratracheal EVs administration. Protective effects of systemically administered MSC-EVs have been described in several models of brain injury, although the mechanisms of action remain undefined.^47-54^ However, in line with our findings, a recent report demonstrated that intratracheal EVs administration is associated with decreased brain inflammation in a murine model of BPD, even if tracing experiments suggested that only a minor proportion of the administered nanoparticles crossed the BBB.^46^ Indeed, it is still a matter of debate whether the immunomodulatory activity of MSC-EVs is mediated by systemic effects, or it requires localization at the site of injury.^50,55^ These findings deserve further investigation, since the choice of the way of administration has important clinical implications both in terms of safety and of efficacy.

Our in vivo model could recapitulate some of the key phases underlying fibrosis in BPD, with the involvement of macrophages. The relationship between oxidative stress and fibrosis seems to be confirmed also in the in vitro model that we adapted from Tang and colleagues.^25^ It is known that the expression of *Nrf2*, a transcription factor of antioxidant genes and a repressor of inflammation,^56^ is present at low levels in myofibroblasts.^57^ In vitro at day 2, macrophages treated with TGFβ alone expressed *Nrf2*, while *α-SMA* gene, that characterizes myofibroblasts, is almost absent. From the early time point, TGFβ acts as pro-oxidative stimulus when added to the macrophages. Importantly, MSC-EVs influence the commitment of macrophages toward myofibroblasts, reducing αSMA expression, as confirmed by flow cytometry and immunofluorescence. Our work has some limitations. First, the rat model cannot recapitulate the complex pathogenesis of human BPD.^58,59^ However, this model mimics the oxidative stress, which has a critical role in the pathogenesis of the disease and which, according to our results, is significantly decreased by MSC-EVs administration in 2 target organs such as lungs and brain. Second, our dose-response study was limited to in vitro assays. Indeed, the relationship between MSC-EVs dosing and therapeutic effect is unclear. Similar results were obtained in animal models of BPD both with a single and with multiple administrations^3,4,46,60,61^A dose-response effect of MSC-EVs was reported in an animal model of intestinal inflammation.^1,62^ However, a study in a murine GvHD model showed that increasing the minimal effective dose of MSC-EVs did not significantly enhance their therapeutic effect.^63^

Of note, we induced the damage (hyperoxia) to the pup rats at the first day after birth (P1), during the canalicular phase of lung development,^64^ which overlaps with the human 24-38 weeks of gestational age. We can say that our model well reproduces the timing of the onset of BPD. Considering the timing of the development of the disease, our model could be compared only to congenital alveolar dysplasia (CAD). However, there is still an important difference: BPD occurs as result of the damage to the airways because of premature birth and CAD results from an early lung development arrest in the canalicular or early saccular stage.^65^

In conclusion, we have shown that human MSC-EVs administration in a rat model of BPD protects from oxidative stress both lungs and brain, 2 critical organs in the pathogenesis of this disease. These results were associated with improved lung epithelial function and reduced fibrosis, pointing to the pleotropic mode of action of these nanoparticles. Additional studies are required to investigate a possible dose-response effect and to evaluate at molecular level the effects of MSC-EVs on the complex pathways linking oxidative stress and fibrogenesis.

## Supplementary Material

szad070_suppl_Supplementary_Figures_1-5_Tables_S1-S3

## Data Availability

The data underlying this article will be shared on reasonable request to the corresponding author.
