# Supplementary material for: Extracellular Vesicles From Mesenchymal Umbilical Cord Cells Exert Protection Against Oxidative Stress and Fibrosis in a Rat Model of Bronchopulmonary Dysplasia
Source: Stem Cells Transl Med. 2023 Nov 15;13(1):43–59. doi: 10.1093/stcltm/szad070 (PMC10785219; doi:10.1093/stcltm/szad070)
Supplement: szad070_suppl_Supplementary_Figures_1-5_Tables_S1-S3 [file szad070_suppl_supplementary_figures_1-5_tables_s1-s3.pdf]

Supplementary information
Bisaccia et al.

Supplementary 1

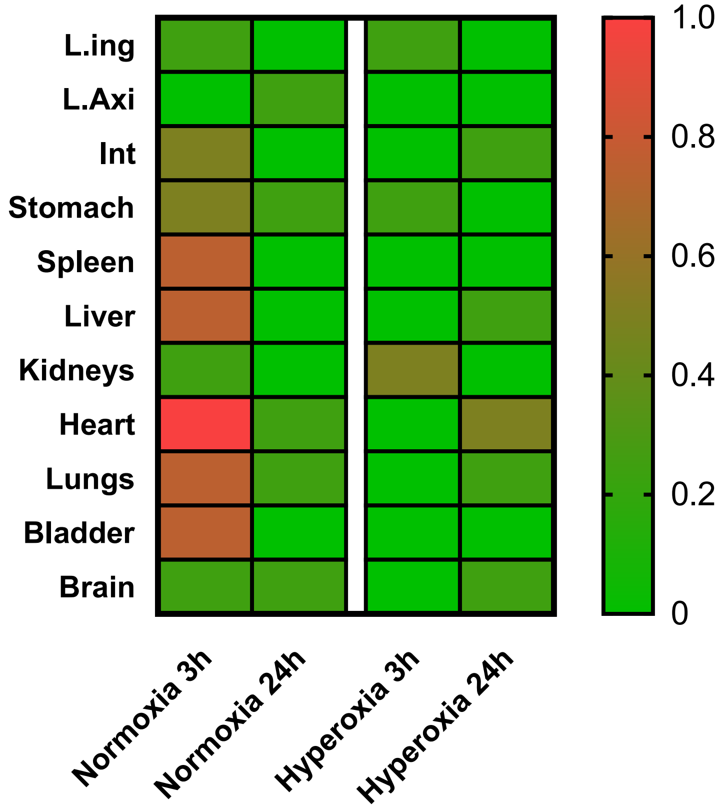

Supplementary 1. Biodistribution at 3 and 24 hours post injection in normoxia and hyperoxia conditions in all organs.

Supplementary 2

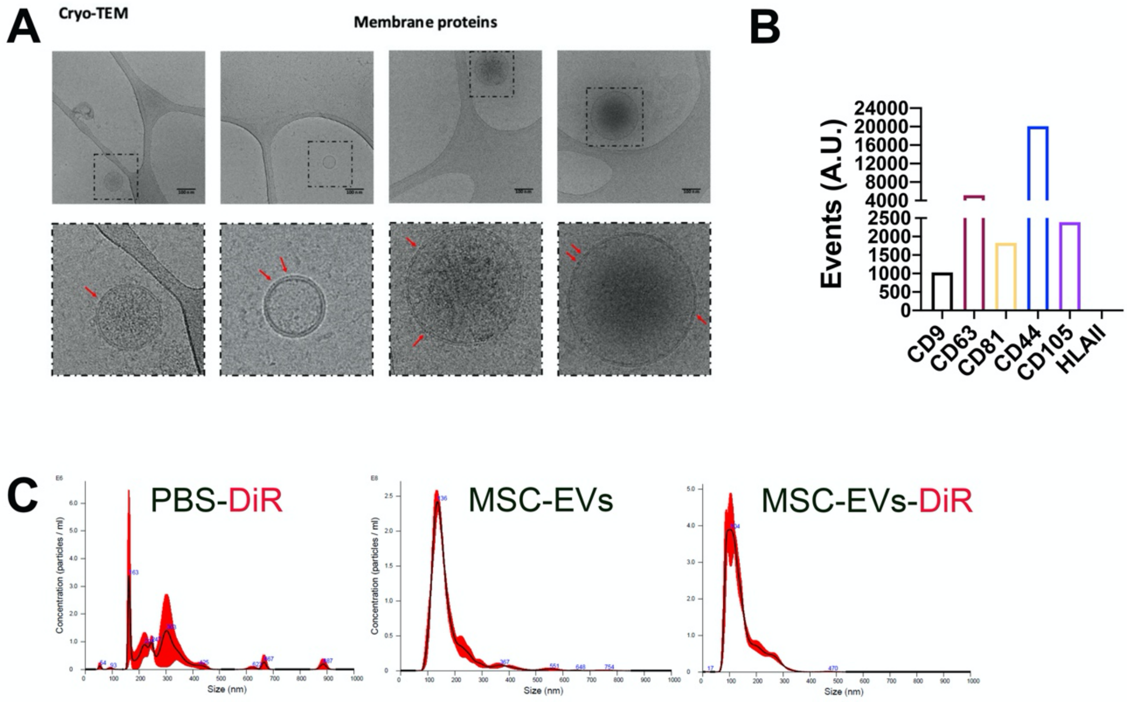

Supplementary 2. A. Cryo TEM of MSC-EVs. **B.** Cytofluorimetric analysis of markers present in MSC-EVs. **C.** NTA analysis of MSC-EVs before and after DiR staining.

Supplementary 3

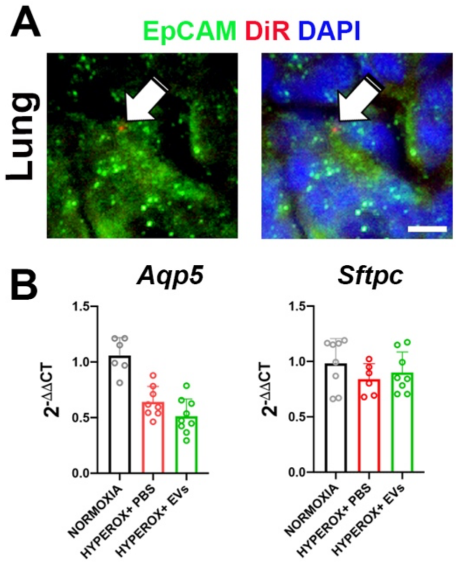

Supplementary 3. A. EpCAM marker and DiR staining. Representative image among a total of 50. Scale bar: 25mm. B. qRT-PCR for Aquaporin 5 (AQP5) and surfactant (SFTPC) genes in lung samples.

Supplementary 4

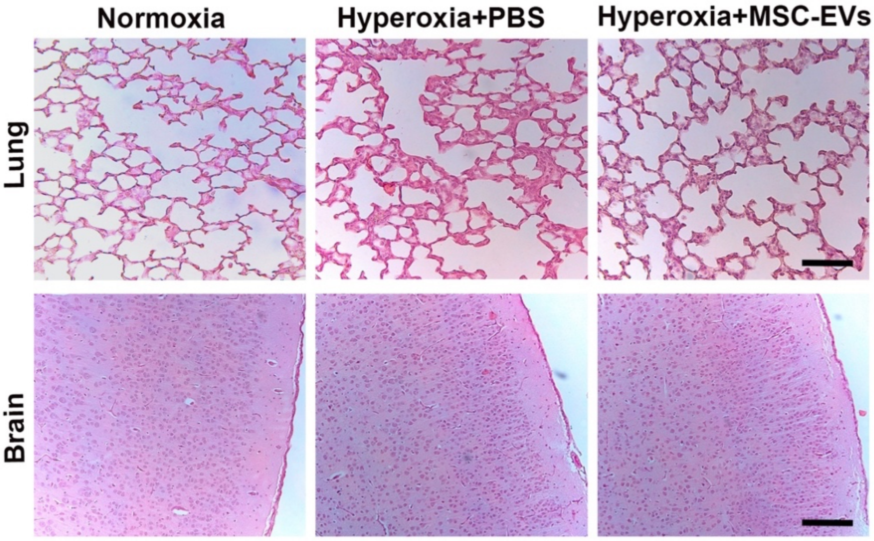

Supplementary 4. Hematoxylin and Eosin in lung and brain. Scale bar for lung: 100μm, for brain: scalebar 200 μm.

Supplementary 5

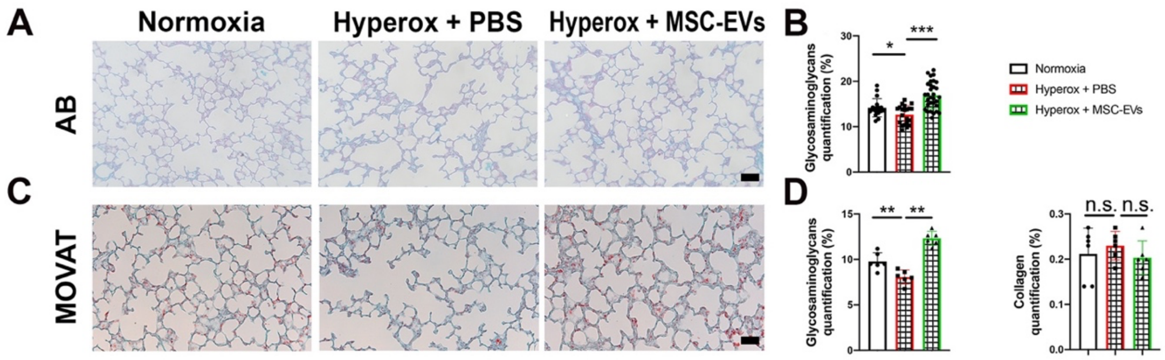

Supplementary 5 . Extracellular matrix protein evaluation: glycosaminoglycans and collagen. A. Alcian Blue staining (AB). **B** Alcian Blue quantification**.** Scale bar 25μm. **C.** Movat staining for hyaluronic acid evaluation. **D.** Hyaluronic acid (blue area) and collagen (yellow area) quantification .

Table S1. Morphometric parameters of alveolarization

Parameter	Normoxia (N=20)	Hyperoxia (N=17)	Hyperoxia + MSC-EVs (N=19)	*p*
Lung volume (cm^3)	2,27 ± 0,06	2,09 ± 0,09	2,31 ± 0,04	0,052
V_{air} (cm^3)	1,19 ± 0,04	1,00 ± 0,06	1,15 ± 0,04	**0,014**
V_{septa} (cm^3)	0,71 ± 0,02	0,74 ± 0,03	0,77 ± 0,02	0,154
Alveolar suface (m^2)	0,130 ± 0,004	0,095 ± 0,003	0,119 ± 0,003	**0,001**
Mean Intercept lenght (µm)	36,8 ± 0,94	41,8 ± 1,7	38,6 ± 1,2	**0,027**
T_{septa} (µm)	10,9 ± 0,3	15,6 ±0,7	13,1 ± 0,4	**0,001**
Alveoli (millions)	5,93 ± 0,2	4,05 ± 0,3	5,80 ± 0,2	**0,001**
Alveolar volume ($µm^3$)	205104 ± 9102	285388 ± 28859	202944 ± 11130	**0,002**

Values are mean ± SEM. P<0,05 to one-way ANOVA are shown in bold

Table S2. Multiple comparisons (Bonferroni's test)

Parameter	Group 1	Group 2	p
V_{air}	Normoxia	Hyperoxia	**0.014**
	Normoxia	Hyperoxia+EV	1.000
	Hyperoxia	Hyperoxia+EV	0.092
Alveolar surface	Normoxia	Hyperoxia	**0.001**
	Normoxia	Hyperoxia+EV	0.099
	Hyperoxia	Hyperoxia+EV	**0.001**
Mean intercept length	Normoxia	Hyperoxia	**0.023**
	Normoxia	Hyperoxia+EV	0.938
	Hyperoxia	Hyperoxia+EV	0.263
T_{septa}	Normoxia	Hyperoxia	**0.001**
	Normoxia	Hyperoxia+EV	**0.007**
	Hyperoxia	Hyperoxia+EV	**0.002**
Alveoli	Normoxia	Hyperoxia	**0.001**
	Normoxia	Hyperoxia+EV	1.000
	Hyperoxia	Hyperoxia+EV	**0.001**
Alveolar volume	Normoxia	Hyperoxia	**0.005**
	Normoxia	Hyperoxia+EV	1.000
	Hyperoxia	Hyperoxia+EV	**0.005**

Table S3. ANOVA test for the values of the graph in panel B figure 8.

Tukey's multiple comparisons test	Significant?	Summary	Adjusted P Value
**DAY6**			
CTRL vs. H_2O_2	Yes	****	<0,0001
CTRL vs. H_2O_2 + VIT C 50µM	Yes	****	<0,0001
CTRL vs. H_2O_2 + VIT C 100µM	Yes	*	0,0486
CTRL vs. H_2O_2 + MSC-EVs 1E8	No	ns	0,7349
CTRL vs. H_2O_2 + MSC-EVs 1E9	Yes	*	0,0481
H_2O_2 vs. H_2O_2 + VIT C 50µM	No	ns	0,9987
H_2O_2 vs. H_2O_2 + VIT C 100µM	No	ns	0,113
H_2O_2 vs. H_2O_2 + MSC-EVs 1E8	Yes	***	0,0005
H_2O_2 vs. H_2O_2 + MSC-EVs 1E9	No	ns	0,1139
H_2O_2 + VIT C 50µM vs. H_2O_2 + VIT C 100µM	No	ns	0,0599
H_2O_2 + VIT C 50µM vs. H_2O_2 + MSC-EVs 1E8	Yes	***	0,0002
H_2O_2 + VIT C 50µM vs. H_2O_2 + MSC-EVs 1E9	No	ns	0,0604
H_2O_2 + VIT C 100µM vs. H_2O_2 + MSC-EVs 1E8	No	ns	0,6507
H_2O_2 + VIT C 100µM vs. H_2O_2 + MSC-EVs 1E9	No	ns	>0,9999
H_2O_2 + MSC-EVs 1E8 vs. H_2O_2 + MSC-EVs 1E9	No	ns	0,6485
**DAY8**			
CTRL vs. H_2O_2	Yes	****	<0,0001
CTRL vs. H_2O_2 + VIT C 50µM	Yes	****	<0,0001
CTRL vs. H_2O_2 + VIT C 100µM	Yes	****	<0,0001
CTRL vs. H_2O_2 + MSC-EVs 1E8	Yes	****	<0,0001
CTRL vs. H_2O_2 + MSC-EVs 1E9	Yes	****	<0,0001
H_2O_2 vs. H_2O_2 + VIT C 50µM	No	ns	0,0705
H_2O_2 vs. H_2O_2 + VIT C 100µM	Yes	**	0,0021
H_2O_2 vs. H_2O_2 + MSC-EVs 1E8	No	ns	0,9336
H_2O_2 vs. H_2O_2 + MSC-EVs 1E9	No	ns	0,983
H_2O_2 + VIT C 50µM vs. H_2O_2 + VIT C 100µM	No	ns	0,6719
H_2O_2 + VIT C 50µM vs. H_2O_2 + MSC-EVs 1E8	No	ns	0,8909
H_2O_2 + VIT C 50µM vs. H_2O_2 + MSC-EVs 1E9	Yes	*	0,0239
H_2O_2 + VIT C 100µM vs. H_2O_2 + MSC-EVs 1E8	No	ns	0,2562
H_2O_2 + VIT C 100µM vs. H_2O_2 + MSC-EVs 1E9	Yes	***	0,0007
H_2O_2 + MSC-EVs 1E8 vs. H_2O_2 + MSC-EVs 1E9	No	ns	0,7238
**DAY10**			
CTRL vs. H_2O_2	Yes	****	<0,0001
CTRL vs. H_2O_2 + VIT C 50µM	Yes	**	0,0014
CTRL vs. H_2O_2 + VIT C 100µM	Yes	****	<0,0001
CTRL vs. H_2O_2 + MSC-EVs 1E8	No	ns	0,455
CTRL vs. H_2O_2 + MSC-EVs 1E9	No	ns	0,9869
H_2O_2 vs. H_2O_2 + VIT C 50µM	Yes	***	0,0007
H_2O_2 vs. H_2O_2 + VIT C 100µM	No	ns	0,7668
H_2O_2 vs. H_2O_2 + MSC-EVs 1E8	Yes	****	<0,0001
H_2O_2 vs. H_2O_2 + MSC-EVs 1E9	Yes	****	<0,0001
H_2O_2 + VIT C 50µM vs. H_2O_2 + VIT C 100µM	Yes	*	0,0444
H_2O_2 + VIT C 50µM vs. H_2O_2 + MSC-EVs 1E8	No	ns	0,0923

H_2O_2 + VIT C 50μM vs. H_2O_2 + MSC-EVs 1E9	Yes	****	<0,0001
H_2O_2 + VIT C 100μM vs. H_2O_2 + MSC-EVs 1E8	Yes	****	<0,0001
H_2O_2 + VIT C 100μM vs. H_2O_2 + MSC-EVs 1E9	Yes	****	<0,0001
H_2O_2 + MSC-EVs 1E8 vs. H_2O_2 + MSC-EVs 1E9	Yes	*	0,0279
